# Pyrosequencing Reveals the Predominance of *Pseudomonadaceae* in Gut Microbiome of a Gall Midge

**DOI:** 10.3390/pathogens3020459

**Published:** 2014-06-11

**Authors:** Raman Bansal, Scot H. Hulbert, John C. Reese, Robert J. Whitworth, Jeffrey J. Stuart, Ming-Shun Chen

**Affiliations:** 1 Department of Entomology, Kansas State University, Manhattan, KS 66506, USA; E-Mails: bansal.67@osu.edu (R.B.); jreese@ksu.edu (J.C.R.); jwhitwor@ksu.edu (R.J.W.); 2 Department of Plant Pathology, Washington State University, Pullman, WA 99164, USA; E-Mail: scot_hulbert@wsu.edu; 3 Department of Entomology, Purdue University, West Lafayette, IN 47907, USA; E-Mail: stuartjj@purdue.edu; 4 USDA-ARS, Hard Winter Wheat Genetics Research Unit, 4008 Throckmorton Hall, Kansas State University, Manhattan, KS 66506, USA

**Keywords:** Hessian fly, *Mayetiola destructor*, gut, microbe, symbiosis

## Abstract

Gut microbes are known to play various roles in insects such as digestion of inaccessible nutrients, synthesis of deficient amino acids, and interaction with ecological environments, including host plants. Here, we analyzed the gut microbiome in Hessian fly, a serious pest of wheat. A total of 3,654 high quality sequences of the V3 hypervariable region of the 16S rRNA gene were obtained through 454-pyrosequencing. From these sequences, 311 operational taxonomic units (OTUs) were obtained at the ≥97% similarity cutoff. In the gut of 1st instar, otu01, a member of *Pseudomonas*, was predominant, representing 90.2% of total sequences. otu13, an unidentified genus in the Pseudomonadaceae family, represented 1.9% of total sequences. The remaining OTUs were each less than 1%. In the gut of the 2nd instar, otu01 and otu13 decreased to 85.5% and 1.5%, respectively. otu04, a member of *Buttiauxella*, represented 9.7% of total sequences. The remaining OTUs were each less than 1%. In the gut of the 3rd instar, otu01 and otu13 further decreased to 29.0% and 0%, respectively. otu06, otu08, and otu16, also three members of the Pseudomonadaceae family were 13.2%, 8.6%, and 2.3%, respectively. In addition, otu04 and otu14, two members of the Enterobacteriaceae family, were 4.7% and 2.5%; otu18 and otu20, two members of the Xanthomonadaceae family, were 1.3% and 1.2%, respectively; otu12, a member of *Achromobacter*, was 4.2%; otu19, a member of *Undibacterium*, was 1.4%; and otu9, otu10, and otu15, members of various families, were 6.1%, 6.3%, and 1.9%, respectively. The investigation into dynamics of *Pseudomonas*, the most abundant genera, revealed that its population level was at peak in freshly hatched or 1 day larvae as well as in later developmental stages, thus suggesting a prominent role for this bacterium in Hessian fly development and in its interaction with host plants. This study is the first comprehensive survey on bacteria associated with the gut of a gall midge, and provides a foundation for future studies to elucidate the roles of gut microbes in Hessian fly virulence and biology.

## 1. Introduction

The gut microbiome consists of microorganisms that live in the digestive tracts of animals, and is an important component of the gut of an organism [1]. There have been numerous studies on the characterization of gut microbiome in insects such as pea aphid, fruit fly, honey bee *etc.* which were reviewed recently [2]. The composition of gut microbiome varies greatly from species to species. Even within the same species, variation in gut microbiome occurs among different individuals, which causes phenotypic differences among these individuals [3]. Diverse microbiome is expected in the gut of insects since different species live in very different ecological environments, and utilize a wide range of food sources from plant tissues to human blood [4]. The insect gut microbes performs a wide range of functions useful to the host, such as synthesizing vitamins and essential amino acids, preventing growth of harmful pathogens, and utilizing energy substrates that cannot be used directly by the host itself [5]. Additionally, insect gut microbes are involved in detoxification of plant secondary metabolites and modification of host plant recognition [6]. Thus, owing to these diverse and vital roles, gut microbes are believed to be key contributors in evolutionary adaptations and ecological success of insects [2].

Gall midges consist of one of the largest and most diversified families in *Insecta* [7]. Most plant-feeding gall midges induce the formation of various types of galls, and many of them are economically important pests in agriculture [8]. So far, no gut microbiota has been systematically characterized from a gall midge. Such studies would provide useful information for a more comprehensive understanding of the biology and ecology of these insects. Hessian fly, *Mayetiola destructor*, is a gall midge and one of the most destructive pests of wheat [9,10]. After hatching, neonatal larvae migrate along the leaf and between leaf-sheaths all the way down to the base of a plant [7]. A first instar larva establishes a permanent feeding site between leaf-sheaths and induces the formation of nutritive cells at the feeding site [11]. The larva stays there and sucks up cell content of nutritive cells until the insect becomes a pupa. The Hessian fly larval gut is one of the most important interfaces for the interaction between the insect and its host plants. There is evidence suggesting that the Hessian fly larval gut contains diverse bacteria such as *Enterobacter*, *Stenotrophomonas*, *Pseudomonas*, and *Bacillus etc.* [12,13,14,15]. Further characterization of the gut microbiota of Hessian fly larvae will provide useful information for research on the ecological and molecular interactions among the Hessian fly, its host plants, and its symbiotic microorganisms.

Lately, high throughput technologies focusing on bacterial 16S rRNA gene have been applied to analyze insect gut microbiomes [16,17,18]. The 16S rRNA gene is generally highly conserved, but between the highly conserved regions, hypervariable regions exist [19]. A total of nine hypervariable regions, named V1 to V9, are present in the 16S rRNA gene. This genic structure makes the 16S rRNA gene useful for microbial identification and phylogenetic analysis [20]. PCR amplification and subsequent sequence analysis of the 16S rRNA gene makes it possible to identify microbes without culturing them. In general, the sequence information of either V3 or V6 variable regions alone provides sufficient data for phylogenetic analysis to the genus level [21]. In this study, we conducted pyrosequencing of the V3 region (corresponding to residues 341 to 534 of the *Escherichia coli* 16S rRNA gene) of DNA samples extracted from the guts of different instars of Hessian fly larvae and amplified directly using PCR. Further, we determined the population dynamics of *Pseudomonas*, the most abundant genera, in different developmental stages of Hessian fly.

## 2. Results and Discussion

To assess the diversity of microbes and their relative richness in the gut of different instars, a total of 6062 reads were obtained for the V3 region of the 16S rRNA gene through 454-pyrosequencing. After removing low-quality reads, 5522 reads were retained for further analysis. Taxonomic classification using ARB-Silva database placed 612 of the reads to the “cyanobacteria-chloroplast” group. The subsequent blastn search using wheat chloroplast genome (GenBank: KC912694.1) as reference confirmed that these reads were derived from wheat chloroplast 16S DNA in the Hessian fly larval gut obtained during feeding. In addition, 494 reads were placed to the Magnoliophyta group (Supplementary Table S1), which were likely derived from wheat 18S DNA present in Hessian fly larval gut since *Triticum aestivum* is a member of the Magnoliophyta flowering plants. This was confirmed through blastn search as these reads showed 99%–100% identity to wheat mitochondrion genome (GenBank: GU985444.1). Furthermore, 762 reads were assigned to the *Insecta* group (Supplementary Table S2). The subsequent blastn search using hessian fly as reference revealed that these reads were derived from Hessian fly 18S DNA (GenBank: KC177284.1). Apparently, the 341F-529R primer pair amplified both wheat and Hessian fly ribosomal genes due to their degenerate nature.

**Figure 1:**
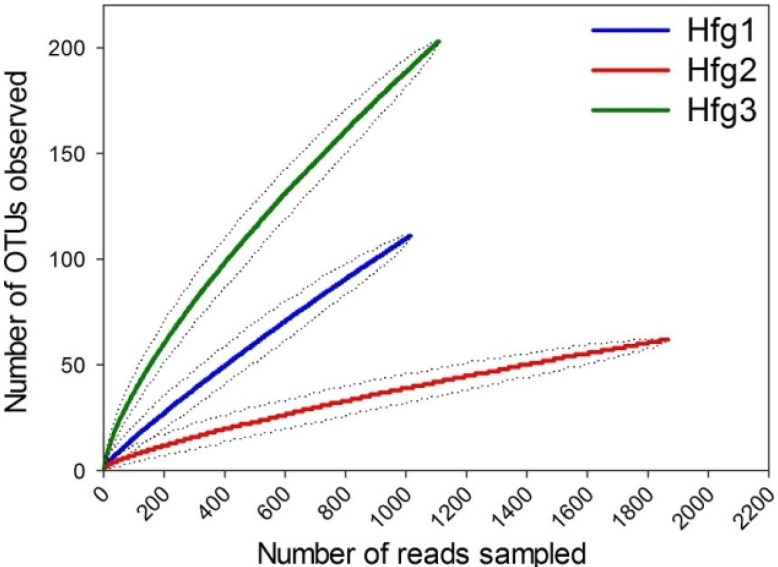
Rarefaction analysis based on resampling without replacement approach.

The remaining 3654 reads was subjected to operational taxonomic unit (OTU) clustering at the ≥97% similarity cutoff (Table 1). The OTU clustering and subsequent rarefaction analysis allowed the comparison of species-richness (number of OTUs) between different larval instars and the determination of adequacy of sequencing output for each stage. The slopes (with respect to *x*-axis) for the Hfg1 and Hfg3 curves were steeper than that from Hfg2 (Figure 1), indicating that for the same number of sequence reads, the 1st and 3rd instar larval guts have much higher richness than the 2nd instar. The rarefaction curve for Hfg2 appeared to reach the plateau/stationary phase (along *x*-axis) but the curves for Hfg1 and Hfg3 did not show proclivity towards *x*-axis. However, the values for “Good’s coverage” for sequencing data in all samples including Hfg1 and Hfg3 (at ≥97% similarity cutoff) ranged between 88.7%–97.7% (Table 1). Taken together, these results suggest that although more species would be expected in the 1st and 3rd instar larval guts with additional sequencing, most bacterial diversity from all samples has already been captured.

**Table 1:** Non-parametric estimates of coverage and species richness.

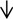 %Similarity ^a^	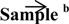	Hfg1	Hfg2	Hfg3
≥99	Coverage (%)	92.0	94.6	81.8
	Ace ^c^	1380.9 (1156.2, 1657.1)	449.2 (331.8, 645.3)	3004.3 (2627.2, 3442.3)
	Chao ^c^	433.1(331.8, 599.9)	410.1(304.9, 590.2)	1284.1 (917.1, 1867.4)
≥97	Coverage (%)	95.4	97.7	87.8
	Ace	440.0 (323.1, 621.1)	201.9 (132.8, 347.2)	1411.7 (1196.9, 1673.2)
	Chao	207.3 (162.9, 289.0)	187.6 (122.8, 332.0)	767.8 (537.6, 1159.2)
≥95	Coverage (%)	96.9	98.5	90.1
	Ace	182.8 (126.7, 299.7)	128.7 (82.9, 234.8)	1132.9 (958.5, 1346.1)
	Chao	153.4 (112.0, 241.2)	103.1 (70.0, 186.2)	521.7 (375.7, 774.5)
≥90	Coverage (%)	98.5	99.2	93.8
	Ace	118.7 (88.6, 170.0)	66.1 (41.6, 138.4)	463.8 (375.6, 582.9)
	Chao	73.6 (56.1,121.4)	80.0 (44.0, 202.5)	281.3 (206.6, 423.0)

^a^ Pyrosequencing reads with a given level of sequence similarity were grouped together; ^b^ Hfg1: first instar larvae gut (1–3 days old); Hfg2: second instar larvae gut (6–8 days old); Hfg3: third instar larvae gut (13–15 days old); ^c^ Ace and Chao are non-parametric measures of species richness. Lower and higher limits (at 95% confidence interval) for these estimates are indicated in parentheses.

Rarefaction curves are shown for operational taxonomic units (OTUs) at ≥97% similarity cutoff for all three samples Hfg1, Hfg2 and Hfg3. Dotted lines represent the upper and lower limits at 95% confidence level. Hfg1: Frist instar larval gut (1–3 days old); Hfg2: Second instar larval gut (6–8 days old); and Hfg3: Third instar larval gut (13–15 days old). The analysis was performed using Mothur [22].

**Figure 2:**
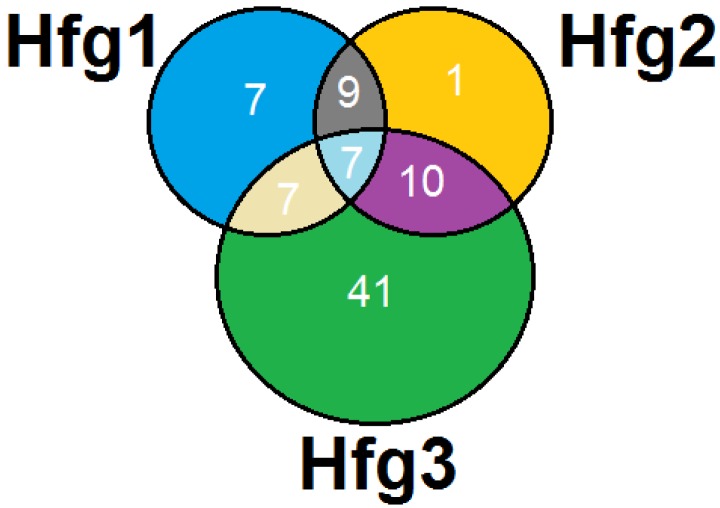
Bacterial OTUs identified from the gut of Hessian fly larvae.

At ≥97% similarity cutoff, the 3654 reads from the three samples were binned into 311 OTUs. Among these OTUs, 229 contained only single reads. These single-read OTUs could either be derived from Hessian fly symbiotic bacteria with very low abundance or due to low amplification efficiency of the 16S DNA from specific bacteria. Alternatively, these single-read OTUs could be due to contamination or sequencing errors during the amplification and sequencing process. The remaining 82 multi-read OTUs (representing 3425 reads, or 93.7% of the total bacterial reads) were subjected to further analysis since they were likely to represent important symbiotic bacteria in the Hessian fly larval gut. As shown in Figure 2, the 82 OTUs were derived from all three samples, but the Hfg3-sample contributed most of them. Fifty-five of the 82 multi-read OTUs, representing 96.53% of the reads contained in these OTUs, were assigned to phylum Proteobacteria, and remaining 27 OTUs were assigned to either Actinobacteria (8 OTUs), Bacteroidetes (3 OTUs), Acidobacteria (2 OTUs), or unclassified (14 OTUs). Among the proteobacterial OTUs, 25 were assigned to Gammaproteobacteria (representing 91.89% of the Proteobacteria reads), 15 to Alphaproteobacteria (5.08% of the Proteobacteria reads), and 14 Betaproteobacteria (2.96% of the Proteobacteria reads), and one unclassified. The Gammaproteobacteria and Actinobacteria reads were found in gut of all three instars whereas Alphaproteobacteria reads were found only in first and third instar stages. However, the Betaproteobacteria and Flavobacteria reads were detected only in the gut of third instar larvae.

Venn diagram showing the number of OTUs (at ≥97% similarity cutoff) that are either unique to or shared between the gut samples. Hfg1: Frist instar larval gut (1–3 days old); Hfg2: Second instar larval gut (6–8 days old); and Hfg3: Third instar larval gut (13–15 days old).

**Figure 3:**
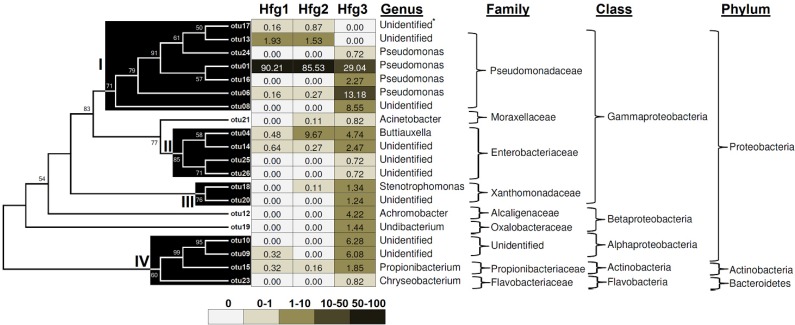
Relative abundance of the top 20 OTUs in different Hessian fly larval instars and their phylogenic relationship.

The phylogenetic relationship and distribution of sequence reads of the 82 OTUs are shown in Supplemental Figure S1. Since the top 20 OTUs represent 94.3% the bacteria reads, we focused on these OTUs for comparative analysis of bacteria abundance and diversity in different Hessian fly larval instars. Phylogenetic grouping resulted in four major clusters (Figure 3). Cluster I, the largest group with seven OTUs and 2713 (or 74.2%) reads, belongs to Pseudomonadaceae. Four of these OTUs, namely otu01, otu06, otu16, and otu24 were identified as *Pseudomonas*, whereas the other three could not be identified to the genus level. otu01 was the most abundant in all three samples. Cluster II, containing four OTUs and 272 (or 7.5%) reads belongs to Enterobacteriaceae. Amongst these OTUs, otu04 was identified as *Buttiauxella*, whereas the other three (otu14, otu25, otu26) could not be identified to the genus level at present. Cluster III consists of two OTUs, otu18 (12 reads, *Stenotrophomonas*) and otu20 (15 reads, unclassified). Cluster IV contains four OTUs and 153 reads, which have diverse bacterial lineages. In cluster IV, otu23 was identified as *Chrysobacterium* (Bacteroidetes), otu15 as *Propionibacterium* (Actinobacteria), and otu09 and otu10 were identified as Rhizobiales (Alphaproteobacteria). Three of the 20 abundant OTUs, otu21 (*Acinetobacter*, Moraxellaceae), otu12 (*Achromobacter*, Alcaligenaceae), and otu19 (*Undibacterium*, Oxalobacteriaceae), could not be placed into any of the major clusters.

Relative abundance (%) of an OTU in a sample was calculated by dividing the number of the corresponding OTU reads with the total bacterial reads. Levels of relative abundances are illustrated by a color scale standing for 0%, 0%–1%, 1%–10%, 10%–50%, and 50%–100%, respectively. Representative sequence for each of top 20 OTUs was used to deduce the phylogenetic inference through Maximum Likelihood method based on Tamura-Nei model [23] in MEGA5.05 [24]. Percentages of replicate trees in which OTU sequences clustered together in the bootstrap test (1000 replicates) are shown (only above 50%) next to the branches in unrooted phylogenetic tree [25]. Taxonomic assignments at genus, class, family, and phylum levels are indicated [*otu17 was not classifiable beyond domain (bacteria) level]. Hfg1: First instar larval gut (1–3 days old); Hfg2: Second instar larval gut (6–8 days old); and Hfg3: Third instar larval gut (13–15 days old).

The composition of gut bacteria varied in different larval instars and diversity increased in later larval stages. In the gut of the 1st instar, 90.2% sequences belong to a single OTU, otu01. The second most abundant OTU, otu13 (also a member of *Pseudomonas*), contained 1.9% sequences; and the remaining OTUs each contained less than 1% sequences. The composition of gut bacteria changed significantly in the 2nd instar. otu01 was still the most abundant in the gut of 2nd instar larvae, but decreased to 85.5%. otu13 also decreased to 1.5%. otu04, on the other hand, increased dramatically from less than 1% in 1st instar to 9.7% in 2nd instar. otu04 belongs to *Buttiauxella* in the Enterobacteriaceae family. The gut bacteria in 3rd instar larvae were the most diversified. Again, otu01 was still the most abundant in the gut of 3rd instar, but decreased to only 29.0%. Otu06 (also *Pseudomonas*) became the second most abundant unit, representing 13.2% of the sequences. In addition, 11 more OTUs were with relative abundance more than 1%, including otu04 (*Buttiauxella*, 4.7%), otu08 (a member of the Pseudomonadaceae, 8.6%) otu09 (a member of Alphaproteobacteria, 6.1%), otu10 (a member of Alphaproteobacteria, 6.3%), otu12 (*Achromobacter*, 4.2%), otu14 (a member of the Enterobacteriaceae, 2.5%), otu15 (Propionibacterium, 1.9%), otu16 (*Pseudomonas*, 2.3%), otu18 (Stenotrophomonas, 1.3%), otu19 (*Undibacterium*, 1.4%), and otu20 (a member of the Xanthomonadaceae, 1.2%). The otu21 (*Acinetobacter*) had relative abundance of less than 1% in both Hfg2 and Hfg3 samples. The OTUs otu08, otu09, otu10, otu12, otu16, otu20, otu23, otu24, otu25, and otu26, were detected only in the Hfg3 sample.

In summary, pyrosequencing revealed *Pseudomonas* as the most abundant genus in the gut of all Hessian fly larval instars. *Pseudomonas* corresponding to otu01 exhibited the highest frequency, but its relative abundance decreases as larvae developed into later instars. On the other hand, *Pseudomonas* bacteria corresponding to otu06 increased in abundance from less than 1% in the gut of 1st and 2nd instars to more than 13% in the gut of 3rd instar. In addition to *Pseudomonas*, *Buttiauxella* was the second most abundant bacteria in the 2nd instar, and remained relatively high abundance in the 3rd instar. Among the three larval instars, the gut of the 3rd instars exhibited the highest number of OTUs and highest richness estimates in spite of having fewer sequences, indicating that the third instar larvae contained more diverse microbes in the gut. At the genus level, the gut of 1st instar contained only two different types of bacteria that exceeded 1% in relative abundance, the gut of 2nd instar contained three different types of bacteria that exceeded 1% in abundance, but the gut of 3rd instar contained 13 different types of bacteria that exceeded 1% in abundance. The biological significance of increased diversity in bacterial composition in 3rd instar larvae remain to be studied.

**Figure 4:**
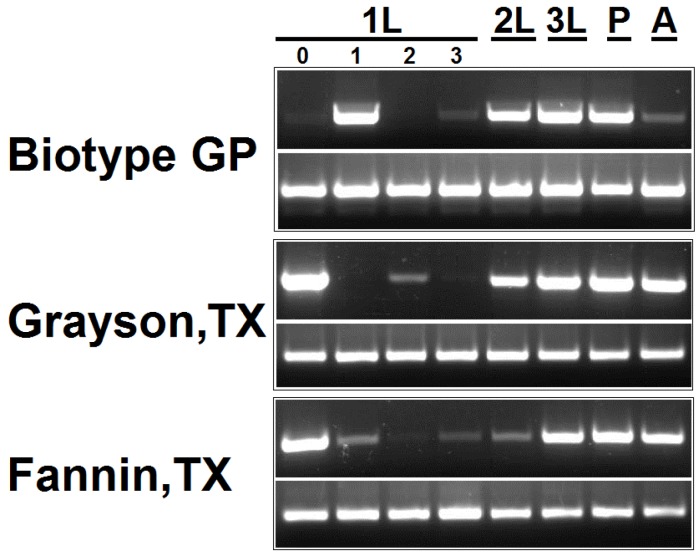
*Pseudomonas* 16S rDNA levels in Hessian fly developmental stages.

As *Pseudomonas* was found to be the most dominant bacteria genera in the gut of Hessian fly larvae, we determined its population dynamics during insect’s various developmental stages including pupae and adults. Measurement of population levels is a step toward its functional characterization as the dynamics curve of a bacterium emphasizes its importance in a particular developmental stage of the insect host. To this end, the relative abundance of *Pseudomonas* 16S rDNA in Hessian fly’s developmental stages is shown in Figure 4. In the first instar larvae, peak level of 16S rDNA was observed on day 0 (in biotype GP) or day 1 (in field populations from Grayson, TX and Fannin, TX, USA); but it fell sharply thereafter during the same instar stage. However during the later developmental stages, high levels of 16S rDNA were observed consistently except in biotype GP adults (females). Higher abundance of *Pseudomonas* in freshly hatched or 1 day old larvae of Hessian fly assumes significance as the *early* first instar larvae of Hessian fly (and other gall midges) is a critical stage that determines the compatibility of the interaction with the wheat (host) plant [26,27]. As opposed to laboratory reared populations, the field populations of Hessian fly feed on diverse wheat cultivars and are continuously exposed to a variety of biotic and abiotic stresses. As a result, field populations experience selection pressures due to different nutritive qualities of wheat varieties, various management practices (such as use of insecticides and cultivation of resistant wheat), natural enemies (pathogens, predators and parasitoids), and other environmental conditions which ultimately can alter the frequency of gut microbes. It is possible that because of the benefits imparted by *Pseudomonas* to adult flies in the field, this bacterium is maintained at higher frequency compared to that in laboratory adults (Figure 4). Thus, it was perhaps not surprising to see different titers of *Pseudomonas* in adults of laboratory (biotype GP) and field (Grayson and Fannin) populations.

Results of semi-quantitative PCR in different developmental stages of biotype GP and of two field populations (Grayson, TX, USA, and Fannin, TX, USA) are shown. For each panel, upper gel is for *Pseudomonas* (33 cycles; 401 bp product) and lower gel is for Hessian fly actin gene (30 cycles: 740 bp product). Figures on the top refer to various developmental stages *i.e.*, 1L: first instar larvae; 2L: second instar larvae (8 days old); 3L: third instar larvae (14 days old); P: pupae; and A: adults. In first instar larval stage, *Pseudomonas* 16S rDNA levels were estimated at four different time points *i.e.*, 0: freshly hatched; 1: one day old; 2: two days old; and 3: three days old.

Earlier, *Pseudomonas* has been found in gut of dipteran flies and other insects [28,29,30,31,32,33,34]; however, the definite evidence on its role in insect biology is lacking. The *Pseudomonas* from olive fly gut hydrolyzes the proteins of olive flesh, suggesting that the olive fly is dependent upon its symbiont for the utilization of its plant [35]. Hessian fly is a member of the gall midges. Galling insects induce the formation of various types of galls [8], a process that requires reprogramming of biochemical and physiological pathways of host plants. The fact that similar bacteria exist in Hessian fly-infested wheat indicates that Hessian fly larvae transmit symbiotic bacteria into host plant tissue during feeding [15]. These results suggest that microbes associated with Hessian fly may play roles both in the physiological process of the insect development and in the interaction with host plants. So far, Hessian fly is the only gall midge in which bacterial association is reported [12,13,14,15]. This research is the first to systematically analyze the microbial community in a galling insect. The discovery of diverse microbes in the gut of Hessian fly larvae should provide a foundation for future research to characterize their functions, and to stimulate similar studies on other galling insects.

## 3. Experimental Section

### 3.1. Insect and Infestation

Larval samples used for pyrosequencing were the progeny of a fly colony that originated from a field infestation collected in Kansas [7]. The majority (99%) of the insects in colony were biotype GP (the Great Plains) although biotypes A, B and others were also found in low frequencies [36]. The susceptible wheat cultivar Karl 92 was used for insect rearing. Plants were grown in environmental chambers at 20 °C with a 12:12 (L:D) photoperiod. Seedlings at 1.5 leaf-stage (full grown first leaf with second leaf just emerged) were infested with Hessian fly eggs by confining adult flies close to plants with a cage with mesh screen. Average egg density was approximately 8 per plant, resulting in approximately 6 larvae per plant.

To estimate population dynamics of *Pseudomonas*, various developmental stages of Hessian fly were collected from first generation of populations which were established from samples collected in Grayson and Fannin counties, TX, where large infestations were present [37]. Populations from field collections were established by following the protocol as described above.

### 3.2. Gut Tissue and DNA Preparation

Hessian larval gut consists of a very small foregut, a major midgut, and a very small hindgut [38]. Gut tissues (containing all the three parts of the gut) were obtained from first (1–3 days old), second (6–8 days old), and third (13–15 days old) instar larvae, respectively. Two hundred guts each from first and second instars, and 100 guts from third instars, were prepared by dissecting larvae under a dissecting microscope. The dissected guts were immediately put into TE buffer (pH 7.5) and homogenized by using a pellet pestle and electric drill for about 20 s/sample. Genomic DNA was extracted by Cetyl trimethylammonium bromide (CTAB) DNA extraction method [39]. Briefly, 250 μL of tissue homogenates were incubated with 500 μL of 2× CTAB buffer (100 mM Tris-HCl, pH 8, 1.4 M NaCl, 20 mM EDTA, 2% (*w*/*v*) CTAB and 0.2% (*v*/*v*) β-mercaptoethanol) at 65 °C for 30 min. The DNA was then extracted using the phenol-chloroform method as described by [40] and precipitated using isopropanol. The DNA pellet was resuspended in 50 μL of nuclease-free water and quantified using a NanoDrop-1000 spectrophotometer (Thermo Scientific, Wilmington, DE, USA).

Three reverse PCR primers, U529R-FC-A33, U529R-FC-A40, U529R-FC-A90 were synthesized for amplifying V3 region of microbial 16S rRNA genes. Each reverse primer consisted of an added sequence for sequencing (primer A, *italic*), a unique barcode (underlined), and a 16S-specific sequence (**bold**). Each reverse primer was used for PCR for a different sample, namely Hfg1, Hfg2, or Hfg3. Hfg1, Hfg2, and Hfg3 were derived from the gut of the first, second, and third instar larvae, respectively. The sole forward PCR primer, U341F-FC-B, consisted of an added sequence (primer B) and a 16S-specific sequence (**bold**).

### 3.3. Template Preparation and Pyrosequencing

To construct bacterial libraries for pyrosequencing, the forward primer U341F-FC-B and one of the three reverse primers (Table 2) were used to amplify the V3 variable region of the 16S rRNA gene from different DNA samples. Specifically, the primer with the barcode TGATG was used to amplify gut DNA from the first instar larvae (Hfg1), the primer with the barcode TCACT was used to amplify gut DNA from the second instar larvae (Hfg2), whereas the primer with the barcode ATACG was used to amply gut DNA from the third instar larvae (Hfg3) as described previously [41]. The samples were amplified in a 25 μL mixture containing 1 μL (10 ng/μL) of DNA as template, 12.5 μL of 2× PCR master mix from Promega (with a final concentration of 0.4 mM each deoxynucleoside triphosphate, 1.5 mM MgCl_2_ and 0.625 units of Taq DNA polymerase in PCR reaction buffer pH 8.5), and 0.32 mM (1 μL) each primer. The reactions were performed on a PTC100 Thermal Cycler (MJ Research, Watertown, MA, USA). The reaction cycle included an initial denaturation at 95 °C for 5 min, followed by 30 cycles of 30 s each at 95, 55, and 72 °C, with a final extension of 5 min at 72 °C. After amplification, the PCR products were purified using a Qiagen QIAquick PCR purification kit (Qiagen, Valencia, CA, USA) and were quantified using a NanoDrop-1000 spectrophotometer (Thermo Scientific, Wilmington, DE, USA). Equal amounts of the PCR products from different samples were pooled and sequenced from the reverse direction by the 454 Life Sciences Company (Branford, CT, USA).

**Table 2:** Primers for template preparation and pyrosequencing.

Primer Name	Primer Sequence	Targeted Sample
U529R-FC-A33	5'*-GCCTCCCTCGCGCCATCAG*TGATG**ACCGCGGCKGCTGGC**	Hfg1
U529R-FC-A40	5'*-GCCTCCCTCGCGCCATCAG*TCACT**ACCGCGGCKGCTGGC**	Hfg2
U529R-FC-A90	5'*-GCCTCCCTCGCGCCATCAG*ATACG**ACCGCGGCKGCTGGC**	Hfg3
Primer A	5'*-GCCTCCCTCGCGCCATCAG*	
U529R	5'-**ACCGCGGCKGCTGGC**	
U341F-FC-B	5'-*GCCTTGCCAGCCCGCTCAG***CCTACGGGRSGCAGCAG**	
Primer B	5'-*GCCTTGCCAGCCCGCTCAG*	
U341F	5'-**CCTACGGGRSGCAGCAG**	

### 3.4. Sequence Processing and Analysis

Based on the 5'-barcode sequences, raw pyrosequencing data was assigned to each sample and 454 sequencing adapters were removed. Reads were discarded if they were <90 bases or >135 bases, or with one or more undetermined or ambiguous nucleotides, or with no valid barcode. After that, sequences were analyzed using Mothur software [22], based on the Schloss SOP [42]. Reads were aligned to the ARB-Silva database of 16S rRNA sequences [43,44]. Pre-clustering was performed (clustering by 2/100 base difference) to reduce pyrosequencing noise *i.e.*, the potential sequence variation introduced due to sequencing error at the nucleotide level [45]. Chimeric sequences were identified and removed using Perseus which takes into account the relative abundance [46]. Remaining reads were assigned into OTUs at different similarity cutoffs (≥90%, ≥95%, ≥97%, ≥99%) using “average neighbor” algorithm. OTUs (assigned at ≥97% similarity cutoff) encompassing only single read were not analyzed further. Reads in each OTU (assigned at ≥97% similarity cutoff) were classified into various genera (or identifiable taxon) using the Bayesian method (bootstrap limit 80%) [47]. The ARB-Silva database was used as reference for taxonomic classification. Additionally, SINA (SILVA Incremental Aligner) tool was used (employing the “least common ancestor” method) to support taxonomic inferences of “ARB-Silva database” search for OTUs identified as “eukaryota” or “unknown” at kingdom level [48]. The sequence reads matching to cyanobacteria-chloroplast, Magnoliophyta, and *Insecta* groups were further identified through wheat and Hessian fly references using NCBI’s blastn tool. Representative sequence for each of the top 20 OTUs (accounting for 94.34% reads classified to “bacteria” kingdom) was used to deduce the phylogenetic inference in MEGA5.05 [24]. The phylogeny was inferred using the Maximum Likelihood method based on the Tamura-Nei model [23]. The bootstrap test with 1000 replicates was conducted to test the reliability [25]. Initial tree for the heuristic search was obtained by applying the Neighbor-Joining method to a matrix of pairwise distances estimated using the Maximum Composite Likelihood approach. The number of observed OTUs was used as a measure of community richness in the gut of larval instars. Estimates of two non-parametric measures of richness, Ace [49] and Chao [50], were performed in Mothur [22]. Rarefaction analysis and coverage estimation were also performed in Mothur [22]. All sequence data were deposited in the Sequence Read Archive, NCBI under BioProject ID# PRJNA231475 (SRP033878).

### 3.5. Estimation of Pseudomonas 16S rDNA Level in Hessian Fly Developmental Stages

DNA extractions from various developmental stage samples of biotype GP, Grayson, TX and Fannin, TX populations were performed as described above. The *Pseudomonas*-specific primers (forward: TAGAGAGRWGCWYGCTTCTCTTGA and reverse: CAATTACGTATTAGGTAACTGCCC were used. Hessian fly actin gene (accession no. AF017427; forward: ATGTGTGACGACGAAGTTGCT and reverse: GGCAACATACATGGCTGGTG-3') was used as a control for template quality and quantity [51]. The samples were amplified in a 25 μL mixture containing 1 μL (10 ng/μL) template DNA, 12.5 μL 2× PCR master mix from Promega (with a final concentration of 0.4 mM each deoxynucleoside triphosphate, 1.5 mM MgCl_2_ and 0.625 units of Taq DNA polymerase in PCR reaction buffer pH 8.5) and 0.32 mM each primer. The reactions were performed on a PTC100 Thermal Cycler (MJ Research, Watertown, MA, USA) and the reaction cycle included an initial denaturation of 5 min at 95 °C followed by variable numbers of cycles of 30 s at 94 °C, 30 s at 55 °C, and 30 s at 72 °C, with a final extension of 5 min at 72 °C.

## Supplementary Files

Supplementary File 1
